# Berberine Improves Inflammatory Responses of Diabetes Mellitus in Zucker Diabetic Fatty Rats and Insulin-Resistant HepG2 Cells through the PPM1B Pathway

**DOI:** 10.1155/2020/2141508

**Published:** 2020-08-18

**Authors:** Yang Sheng Wu, Zhe Ming Li, Yi Tao Chen, Shi Jie Dai, Xiao Jie Zhou, Yuan Xiao Yang, Jian Shu Lou, Li Ting Ji, Yu Ting Bao, Ling Xuan, Lu Ning Lin, Chang Yu Li

**Affiliations:** ^1^College of Pharmacy, Zhejiang Chinese Medical University, Hangzhou, Zhejiang, China; ^2^College of Life Sciences, Zhejiang Chinese Medical University, Hangzhou, Zhejiang, China; ^3^School of Basic Medical Sciences and Forensic Medicine, Hangzhou Medical College, Hangzhou, Zhejiang, China; ^4^Key Laboratory of Elemene Class Anti-Cancer Chinese Medicine of Zhejiang Province, Zhejiang, China; ^5^Holistic Integrative Pharmacy Institutes, School of Medicine, Hangzhou Normal University, Zhejiang, China

## Abstract

Berberine (BBR), a natural compound extracted from a Chinese herb, has been shown to effectively attenuate insulin resistance (IR) and inflammation in the clinic. However, its ameliorative mechanism against IR is not well defined. This study is aimed at investigating the effect of BBR and protein phosphatase, Mg^2+^/Mn^2+^-dependent 1B (PPM1B) on IR. Biochemical measurements and liver histopathology were detected using the biochemical analyzer and HE staining in ZDF rats, respectively. Microarray analysis of liver tissues was performed, and differentially expressed gene (DEG) levels were examined by quantitative real-time PCR (qPCR) and Western blot. Additionally, the effect of BBR was also explored in HepG2-IR cells. The glucose oxidase method and the fluorescent glucose analog were used to detect glucose consumption and uptake, respectively. The PKA inhibitor H89, ELISA, qPCR, Western blot, and immunofluorescence staining were employed to estimate the expression levels of related signaling pathways. To evaluate the roles of PPM1B, HepG2-IR cells were stably infected with lentivirus targeting PPM1B. The administration of BBR drastically decreased the body weight, urine volume, blood glucose, blood urea nitrogen (BUN), CHOL, hepatic index levels, and pathologic changes and improved ALB levels in ZDF rats with PPM1B upregulation. Furthermore, BBR effectively improves glucose consumption, uptake, and inflammation in HepG2-IR cells. The knockdown of PPM1B expression aggravated the inflammatory response and glycometabolism disorder in HepG2-IR cells. Mechanistically, a reversal in the expression of cAMP, PKA, PPM1B, PPAR*γ*, LRP1, GLUT4, NF-*κ*B p65, JNK, pIKK*β* Ser181, IKK*β*, IRS-1 Ser307, IRS-1, IRS-2 Ser731, IRS-2, PI3K p85, and AKT Ser473 contributes to ameliorate IR in HepG2-IR cells with BBR treatment. Altogether, these results suggest that BBR might regulate IR progression through the regulation of the cAMP, PKA, PPM1B, PPAR*γ*, LRP1, GLUT4, NF-*κ*B p65, JNK, pIKK*β* Ser181, IKK*β*, IRS-1 Ser307, IRS-1, IRS-2 Ser731, IRS-2, PI3K p85, and AKT Ser473 expression in the liver.

## 1. Introduction

Diabetes mellitus (DM) is characterized by deficiency or resistance to insulin in peripheral tissues with persistent hyperglycemia [1], which resulted in increasing healthcare costs globally. People with diabetes are at higher risk of heart, brain, and peripheral vascular diseases by 6-7 times with long-range complications such as neuropathy, retinopathy, and nephropathy [2, 3], and over 90% of all the diagnosed cases are type 2 diabetes (T2D) [4]. From prediabetes to the later stages of T2D, insulin resistance (IR) is associated with the development of T2D and also viewed as an autoinflammatory disease [5, 6]. Previous studies showed that chronic low-grade inflammation is considered to be one of the main causes of T2D [7, 8]. In spite of the efforts that have been made in the treatment for IR, the detailed mechanism has not been fully elucidated.

Traditional Chinese medicine (TCM), as complementary and alternative medicine, has been frequently reported to possess promising effects toward DM and its complications. Recently, Chinese herbs have received more attention as antidiabetes medication. *Rhizoma coptidis* was recorded approximately 1500 years ago in a book titled *Note of Elite Physicians* by Hongjing Tao [4]. Berberine (BBR, [C_20_H_18_NO_4_]C), an isoquinoline alkaloid, extracted from *Berberis vulgaris*, *Cortex phellodendri*, and *Rhizoma coptidis* [9, 10], is characterized by high safety in both animals and humans. It has been clarified that BBR possesses multiple pharmacological activities, including anti-inflammatory [11, 12], antimicrobial, antitumor, and antifibrosis effects [13]. In addition, the effects of BBR on insulin sensitivity and glucose tolerance have shown promising prospects on metabolic disorders [14, 15]. Therefore, determining the mechanism of BBR could be a promising therapeutic strategy for T2D treatment.

In this study, we had a great interest in the protein phosphatase, Mg^2+^/Mn^2+^-dependent 1B (PPM1B) that belongs to Ser/Thr protein phosphatase (PP2C) family [16]. PPM1B is a single subunit enzyme that requires magnesium/manganese for activity with a catalytic domain and regulatory domain [17, 18]. Several kinases, receptors, channels, and transcription factors have been identified to be the substrates for PP2C phosphatases, suggesting that PPM1B participates in diverse physiological effects such as stress response, metabolism, and cell cycle [19]. It is well documented that IKK*β* serves as a central intermediate signaling molecule in TNF-*α*-induced NF-*κ*B activation. Investigations have found that overexpression of PPM1B suppresses the inflammatory signaling and IKK*β* induced NF-*κ*B activation by dephosphorylation of IKK*β* at Ser177 and Ser181 [20, 21]. In addition, the phosphorylation of IKK*β* plays a critical role in the regulation of the insulin signal pathway [21]. Therefore, we speculated that PPM1B-mediated inflammation and regulation of insulin signaling are the significant causes of the initiation and development of T2D. However, the PPM1B-related molecular mechanisms remain a mystery.

Zucker diabetic fatty (ZDF) rats and HepG2-IR cells were used for exploring the effect and mechanism of BBR by targeting PPM1B signaling. Furthermore, PPAR*γ*, LRP1, GLUT4, NF-*κ*B, JNK, IKK*β*, IRS-1, IRS-2, PI3K, and AKT expression in the liver was also detected. Meanwhile, the effect of BBR is explored in vitro on glucose consumption, uptake, and the expression of the inflammatory factor in HepG2-IR cells. Additionally, we tried to clarify whether multiple pathways are associated with PPM1B in HepG2-IR cells. Moreover, to further determine the roles of PPM1B, we silenced PPM1B expression via lentivirus-induced HepG2-IR cells. In the present study, it was found that PPM1B was downregulated in ZDF liver tissues and HepG2-IR cells, and BBR reversed the decrease of PPM1B. In addition, our study consistently demonstrated that BBR improves glucose consumption and uptake and depresses the inflammatory response in HepG2-IR cells. However, few studies have expatiated on whether its therapeutic function is associated with the regulation of the PPM1B, PPAR*γ*, LRP1, GLUT4, NF-*κ*B p65, JNK, and IKK*β*.

The purpose of the current research was to investigate preliminarily whether the molecular mechanism of BBR treats T2D by regulating the expression of PPM1B, PPAR*γ*, LRP1, GLUT4, NF-*κ*B p65, JNK, IKK*β*, IRS-1, IRS-2, PI3K p85, and AKT in the ZDF rats' liver tissue and HepG2-IR cells.

## 2. Materials and Methods

### 2.1. Animal Interventions

42 male ZDF rats (ZDF^fa/fa^/ZDF^fa/+^) were acquired from the Animal Center of Zhejiang Chinese Medicine University at the age of 6 weeks. ZDF^fa/fa^ rats (*n* = 36) were used after administration with a high-fat diet for 7 weeks. The high-fat diet consisted of 59% basis feed with 10% egg yolks, 10% sucrose, 20% lard, and 1% cholesterol, which was provided by the Animal Center of Zhejiang Chinese Medicine University. Meanwhile, ZDF^fa/+^ rats (*n* = 6) received a normal chow at the start of the study. Animals were housed under controlled temperature 22 ± 1°C with 60 ± 5% humidity, a 12 h light-dark cycle, and free access to water and chow. Animals with blood glucose levels consistently greater than 16.7 mmol/L were considered diabetic, and animals were divided into 7 groups (6 animals/group): the control group (ZDF^fa/+^ rats administrated with a control rat diet and an equivalent volume of 0.5% gum tragacanth in normal saline); the model (DM) group (ZDF^fa/fa^ rats fed a high-fat diet); the glimepiride (Gli) group (5 mg/kg/d glimepiride; Sanofi Pharmaceutical Co., Ltd., Beijing, China); the metformin (Met) group (200 mg/kg/d metformin; Sino-American Shanghai Squibb Co., Ltd., Shanghai, China); the combination of Gli and Met group (5 mg/kg/d glimepiride+200 mg/kg/d metformin); the low-dose BBR group (purity > 98%; Xi'an Kai Lai Biological Engineering Co., Ltd., Xi'an, China; T2D rats administrated with 150 mg/kg/d body weight BBR by gavage); and the high-dose BBR group (T2D rats administrated with 300 mg/kg body weight BBR by gavage), which also were treated continuously for 12 weeks. All the experiments were reviewed and approved by the Animal Care and Welfare Committee of Zhejiang Chinese Medical University (permit number: ZSLL-20130-106).

### 2.2. Biochemical Measurements and Histopathology

In the 12^th^ week, following a 12 h fast, blood samples were extracted via tail vein puncturing. Fasting blood glucose (FBG) was detected by a blood glucose meter (ACCU-CHEK® Performa; Roche Co., Ltd., Shanghai, China). For each rat, body weight was examined weekly after treatment. Also, the rats were singly housed in metabolic cages to investigate the food/water intake and urine volume. After that, animals were sacrificed by bleeding from the heart under pentobarbital sodium (45 mg/kg, i.p.) anesthesia, and then blood samples were centrifuged at 3000 rpm/min for 15 minutes at 4°C to collect the sera for the measurement of BUN, CHOL, LDL-C, AST, ALT, and ALB by a fully automatic biochemical analyzer (7020, Hitachi, Japan). The liver tissues were excised and weighed, and the hepatic index (liver weight/body weight ratio) was calculated. Subsequently, the partial liver was frozen at −80°C until further analysis, and the remnant liver was fixed in 4% paraformaldehyde.

For histological examination, the paraformaldehyde-fixed livers were embedded in paraffin and cut into 4 *μ*m sections; the sectioned liver samples were then stained with hematoxylin and eosin. The images were captured and viewed at magnifications of 400x using a DMI3000B light microscope (Leica, Wetzlar, Germany).

### 2.3. Transcriptomic Analysis

Total RNA of the control, DM, and low- and high-dose BBR groups (*n* = 3 per group) were isolated using the TRIzol reagent (Invitrogen, USA) according to the manufacturer's instructions. The RNA concentration was quantified using the NanoDrop ND-2000 (Thermo Fisher Scientific, USA). Transcriptomic analysis was performed by LC-Bio Corporation (Hangzhou, Zhejiang). Briefly, the microarray chips were scanned using Agilent Scanner G2505C (Agilent Technologies, Inc.). The microarray data were extracted by using GenePix® Pro 6.1 (Molecular Devices, LLC, Sunnyvale, CA, USA) and normalized with Agilent_Analyze_V1.0 (Agilent Technologies, Inc.).

### 2.4. Functional Enrichment Analysis and Identified Core Targets

The DEGs between the control, DM, and BBR groups were identified with an absolute log2 (Con/DM) ≥ 1 and log2 (BBR/DM) ≥ 1 and combined with the *P* values < 0.05. To improve the understanding of biological information about the DEGs, we used the Gene Ontology (GO) platform (http://www.geneontology.org) [22], an international standardization of the gene function classification system to describe the molecular function, cellular component, and biological process of DEGs. KEGG pathway enrichment analyses of DEGs were conducted with DAVID (http://david.abcc.ncifcrf.gov/) [23], a tool that provides a comprehensive biological meaning for a large list of genes. False discovery rate (FDR) < 0.05 was the cutoff criterion value.

### 2.5. Effect of BBR on HepG2 Cell Viability

**Figure 1:**
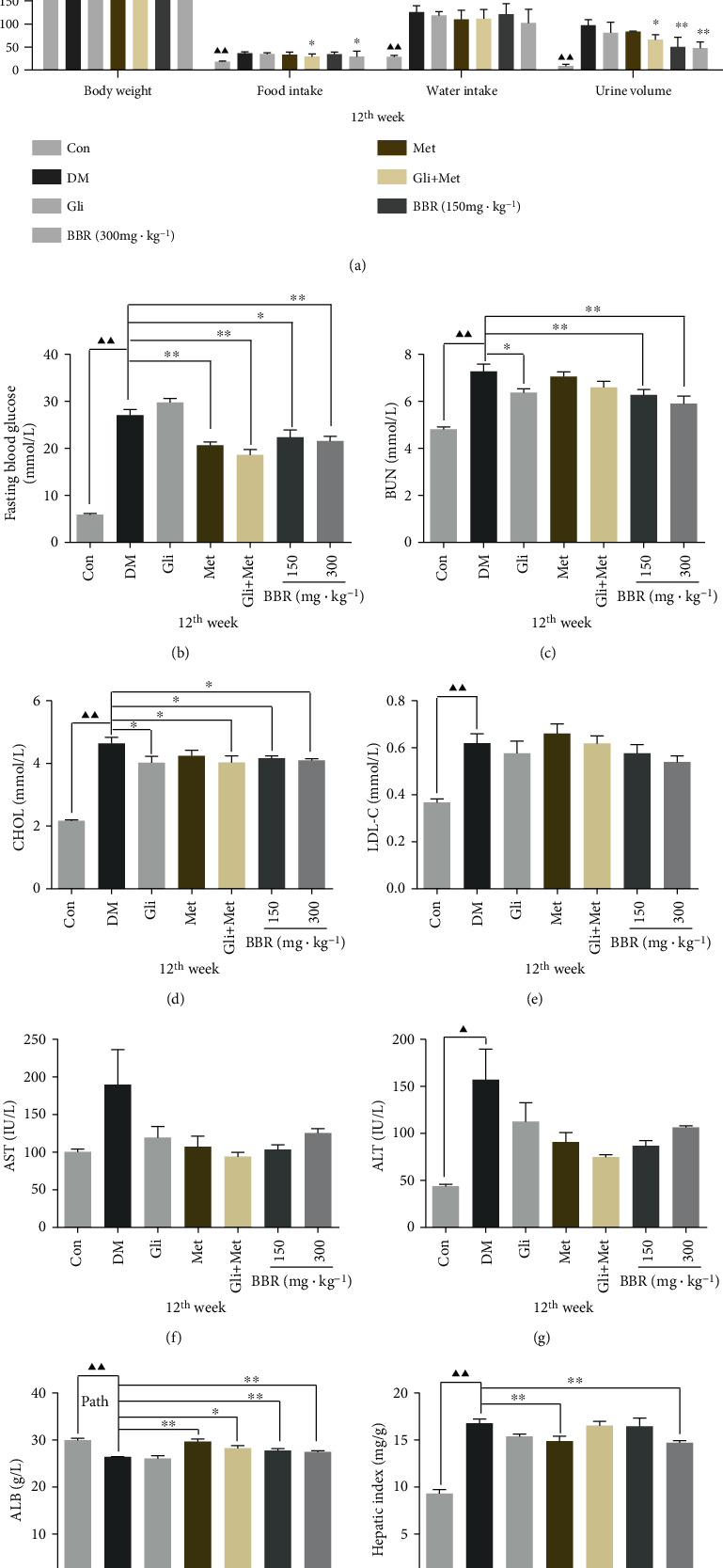
Berberine ameliorates metabolic disorders in ZDF rats. (a) Body weight, food intake, water intake, and urine volume. (b) Fasting blood glucose. Serum levels of (c) BUN, (d) CHOL, (e) LDL-C, (f) AST, (g) ALT, (h) ALB, and (i) hepatic index were evaluated in control rats and ZDF rats with or without glimepiride, metformin, their combination, and BBR treatment. Data are presented as mean ± standard error (*n* = 6 rats for each group). ^▲^*P* < 0.05 and ^▲▲^*P* < 0.01, Con vs. DM; ^∗^*P* < 0.05 and ^∗∗^*P* < 0.01, DM vs. experimental groups. BUN: blood urea nitrogen; CHOL: cholesterol; LDL-C: low-density lipoprotein cholesterol; AST: aspartate aminotransferase; ALT: alanine aminotransferase; ALB: albumin; GLU: glucose.

The HepG2 cells were cultured in Dulbecco's modified Eagle's medium (DMEM, Gibco, USA) supplemented with 10% fetal bovine serum (FBS), 100 *μ*g/mL streptomycin, and 100 IU/mL penicillin at 37°C, 5% CO_2_ atmosphere. 5 × 10^3^ cells/well were placed in a 96-well plate and treated with BBR at a dose of 5, 10, 20, 30, 40, and 50 *μ*g/mL for 24 h or 48 h. 3-(4,5-Dimethylthiazol-2-yl)-2,5-diphenyltetrazolium bromide (MTT, 0.5 mg/mL) in PBS was then added to each well and placed in a spectrophotometer (iMark/xMark; Bio-Rad, Shanghai, China). The cell viability was revealed by the absorbance which was measured at 570 nm. As a result, we discovered that BBR crippled the viability of HepG2 cells at the dosage of 30, 40, and 50 *μ*g/mL. There was no obvious impact of BBR on HepG2 cell viability at the dosage of 5, 10, and 20 *μ*g/mL. These data indicated that BBR at the dosage of 5, 10, and 20 *μ*g/mL had no toxicity to cells. Therefore, we selected 5, 10, and 20 *μ*g/mL of BBR for the subsequent experiments (Figure 1S).

### 2.6. Establishment of the HepG2-IR Model

**Figure 2:**
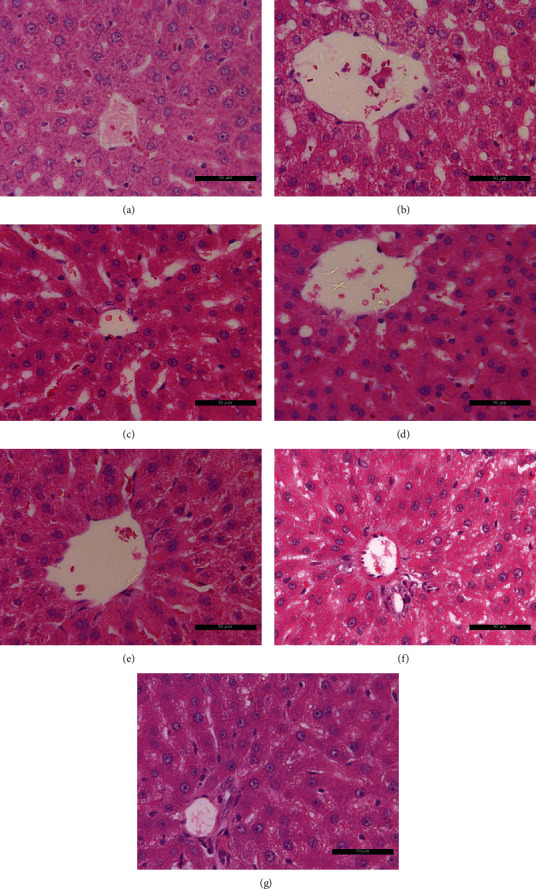
Berberine decreases the hepatic lipid accumulation. (a) Control group, (b) model (DM) group, (c) glimepiride group, (d) metformin group, (e) glimepiride+metformin group, (f) berberine (150 mg/kg) group, and (g) berberine (300 mg/kg) group. Scale bars, 50 *μ*m.

The IR of HepG2 cells was established based on the study of Zhang et al. [24] with modification. Briefly, cells were cultured and serum-starved for 2 h in 96-well plates, and the medium was replaced by serum-free DMEM supplemented with 10^−5^, 10^−6^, 10^−7^, and 10^−8^ mol/L insulin (Sigma) for 36 h. Subsequently, the medium was collected and the glucose content was measured using a glucose test kit (Applygen, China). In addition, glucose uptake was measured using the fluorescent glucose analog, 2-NBDG (2-(N-(7-nitrobenz-2-oxa-1,3-diazol-4-yl)amino)-2-deoxyglucose; Thermo Fisher Scientific). In this study, the glucose consumption and uptake of the 10^−6^ mol/L insulin-treated groups significantly decreased, indicating that the HepG2-IR model was established successfully (Figure 2S).

### 2.7. Glucose Consumption and Uptake

Cells were seeded into 96-well plates with six wells left as blanks. As mentioned above, the HepG2-IR cell model was established. Then, BBR (5, 10, and 20 *μ*g/mL) was applied to each well (six replicates) and incubated for 24 h. Next, the glucose consumption in the medium was calculated with the glucose oxidase method and normalized by the cell number detected by MTT. For glucose uptake assay, cells were cultured and molded in 24-well plates and treated with BBR for 24 h, and a solution of 200 *μ*mol/L 2-NBDG in PBS was applied for 30 min at 37°C. The cell images were obtained using a fluorescence microscope (DMI3000B, Leica, Germany). The mean fluorescence intensity of images was then calculated by Image-Pro Plus 6.0 software (Media Cybernetics, Silver Spring, MD, USA), which represents the glucose uptake.

### 2.8. Detection of cAMP in HepG2-IR Cells

After 24 h of treatment, HepG2-IR cells were lysed on ice for 30 min in RIPA lysis buffer and centrifuged at 3000 rpm for 20 min at 4°C. The cell-lysed supernatants were collected and stored at –20°C for measurement of cAMP by the ELISA kit (Shanghai Xin Fan Biotechnology Co., Ltd., China) according to the manufacturer's instructions.

### 2.9. qPCR

Total RNA of liver tissues and HepG2-IR cells was extracted using the TRIzol reagent kit, according to the manufacturer's instructions. The purity and concentration were assayed with a NanoDrop 2000 (Thermo Fisher Scientific, USA), and cDNA was synthesized using PrimeScript™ RT Master Mix (Perfect Real Time). SYBR Green Master Mix (TaKaRa, Dalian, China) was used to perform qPCR analysis on a CFX96 Touch Deep Well detection system (Bio-Rad, USA). Relative data were normalized to the housekeeping gene glyceraldehyde-3-phosphate dehydrogenase (GAPDH) and calculated by the 2^-*ΔΔ*Ct^ method. Primers involved in this article are shown in Supplementary Table S1.

### 2.10. Western Blotting

Total protein was prepared from liver tissues and HepG2-IR cells using RIPA buffer (Beyotime, China) with protease inhibitor cocktail. The total protein concentration was quantified by the Bicinchoninic Acid Protein Assay Kit (Beyotime, China). An equivalent amount of protein samples were separated by 10-15% sodium dodecyl sulfate-polyacrylamide gel electrophoresis (SDS-PAGE) and transferred to polyvinylidene difluoride (PVDF) membranes (Bio-Rad, USA). Then, the membranes were exposed to 5% skim milk at room temperature for 2 h before incubated with primary antibody and secondary horseradish peroxidase-conjugated antibody (LI-COR, USA). Finally, the protein bands were visualized by the Odyssey® IR scanner (LI-COR, USA) and quantified by the ImageJ software (National Institutes of Health, NY). Furthermore, H89 was combined with BBR (10 *μ*g/mL) to investigate the PPM1B protein level in HepG2-IR cells. To estimate the nuclear translocation of NF-*κ*B p65, the nuclear protein of HepG2-IR cells was extracted by NE-PER Nuclear and Cytoplasmic Extraction Reagents (Thermo Fisher Scientific) according to the manufacturer's protocol.

### 2.11. Immunofluorescence

The HepG2-IR cells treated with BBR were fixed with 4% formaldehyde and permeabilized in 0.5% Triton X-100. The fixed HepG2-IR cells were washed with PBS and blocked with 5% bovine serum albumin in PBS for 60 min at room temperature. Fixed HepG2-IR cells were then incubated with the primary antibodies overnight at 4°C, then incubated with Alexa Fluor 488-conjugated donkey anti-rabbit IgG antibody (1 : 1000; Life Technologies, Waltham, MA, USA) or Alexa Fluor 647-conjugated goat anti-mouse IgG antibody (1 : 200; ab1501115, Abcam, UK) for 1 h at room temperature, followed by DAPI staining (Solarbio Life Sciences, China) in the dark. Images were taken by a fluorescence microscope.

### 2.12. shRNA PPM1B Transfection

**Figure 3:**
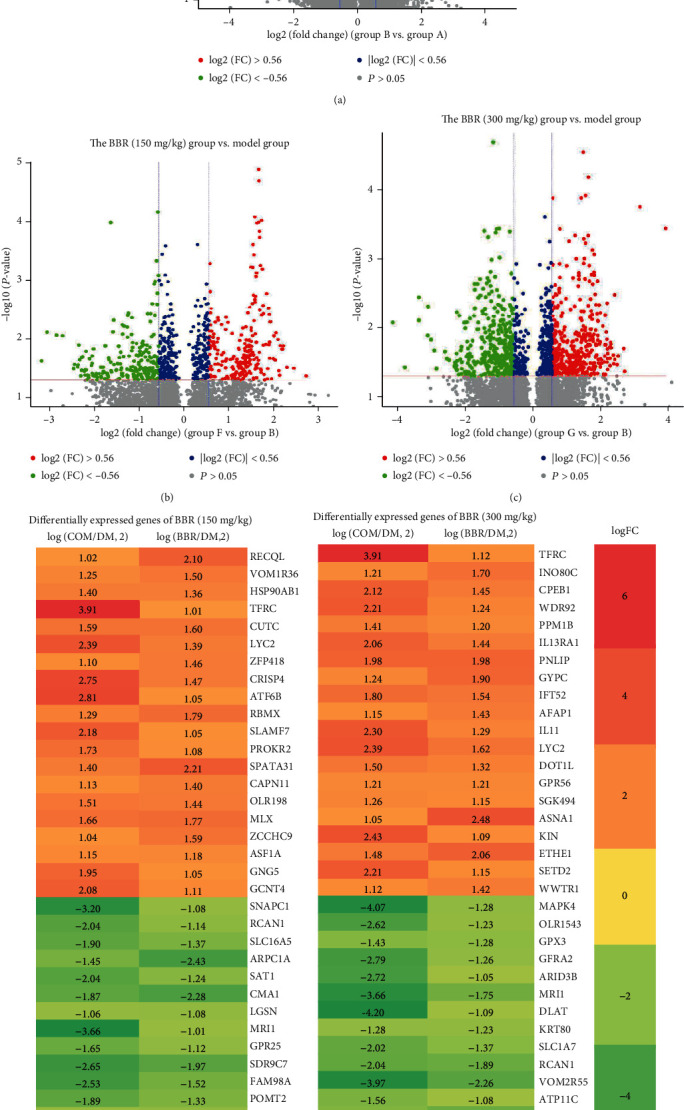
Volcano plot of gene expression profile data between the (a) model and normal samples, (b) BBR (150 mg/kg) and model groups, and (c) BBR (300 mg/kg) and model groups. Heat map of DEGs in (d) BBR (150 mg/kg) and (e) BBR (300 mg/kg). Green represents a lower fold change value; red represents a higher fold change value. Each column represents one dataset, and each row represents one gene. The number in each rectangle represents fold change in the BBR compared with the control samples or the control samples compared with the model samples. The gradual color that ranged from red to green represents the changing process from upregulation to downregulation.

The PPM1B-specific (GenBank accession no. NM_177969) targeting shRNA sequences (5′-GCAAGCGTAATGTTATTGA-3′/shPPM1B-1, 5′-CACGGGTTGAAGAGATTAT-3′/shPPM1B-2, and 5′-CTGAATCCACATAGAGAAA-3′/shPPM1B-3) and a negative control shRNA sequence (5′-TTCTCCGAACGTGTCACGT-3′/shPPM1B-NC) were synthesized by GeneChem (Shanghai, China). For lentiviral infection, Cells were plated into 6-well plates and infected with either shPPM1B (MOI = 10) or shPPM1B-NC lentiviruses in the presence of 5 mg/mL polybrene (Genomeditech, Shanghai, China). After 72 h, the GFP level from the lentivirus in HepG2 cells was assessed using the fluorescence microscope. Then, the cells were harvested and measured for the posttransfection using qPCR and Western blotting. The present results suggested that the PPM1B expression from the viral vector was observed in >90% of HepG2 cells. Further, the qPCR and Western blot assay elucidated that shPPM1B-1/PPM1B-2/PPM1B-3 were effective in HepG2 cells, with approximately 40-60% knockdown efficiency. These data suggested that the lentiviral-mediated shRNA avenue could efficiently and stably weaken the PPM1B level in HepG2 cells, and knockdown efficiency of shPPM1B-1 is especially remarkable that it was chosen for further study (Figure 3S).

### 2.13. Effect of BBR on PPM1B Knockdown HepG2-IR Cells

HepG2 cells were preinfected with scrambled or shRNA PPM1B and treated with BBR after the incubation of insulin for 36 h. Subsequently, cell glucose consumption and uptake were detected. Additionally, shPPM1B- and shNC-infected HepG2 cells (3 × 10^5^ cells/well) were seeded into 6-well plates, to measure the expression of inflammatory cytokines as well as the mRNA and protein expression levels of PPM1B pathways via qPCR and Western blotting analysis as mentioned above.

### 2.14. Statistical Analysis

Quantitative data are expressed as mean ± standard deviation (SD). Student's *t*-test, repeated measurements, and a post hoc analysis (the least significant difference (LSD) test) were used to determine the each variable within and between groups, and the statistical significance was set at *P* < 0.05.

## 3. Results

### 3.1. BBR Improves the Confusion of Metabolism in ZDF Rats

Compared with DM rats, the body weight of the glimepiride, metformin, and their combination groups significantly decreased (*P* < 0.05), the food intake and urine volume were significantly (*P* < 0.05 or *P* < 0.01) lower in the BBR-treated groups, and there were no differences in water intake between different groups (Figure 1(a)). The FBG, BUN, and CHOL levels in ZDF rats were significantly greater than those in the control group (*P* < 0.01) and attenuated by BBR treatment for 12 weeks (*P* < 0.05) (Figures 1(b)–1(d)). No significant changes were found for LDL-C, AST, and ALT but had a decreased tendency in experimental groups (Figures 1(e)–1(g)). Instead, a significant elevation on ALB was observed between the DM and BBR-treated groups (Figure 1(h)). Also, the hepatic index was significantly decreased in the treated groups vs. the DM group (Figure 1(i)). These data indicated that BBR treatment had protection on the liver and kidney and ultimately improved dysfunction in ZDF rats.

### 3.2. Effect of Berberine on Histopathological Changes in the Liver

H&E staining showed larger vacuoles and liver cells arranged neatly in the livers of ZDF rats than in those of control rats (Figure 2(a)), indicating the accumulation of lipid droplets, and the shape of liver cells changed when the volume of liver cells increased (Figure 2(b)). In contrast, glimepiride, metformin, and their combination as well as BBR treatment ameliorated the appearance of large vacuoles and arranged the disorder of liver cells in ZDF rats (Figures 2(c)–2(g)), suggesting BBR has a protective effect on pathological changes in the liver of diabetic rats.

### 3.3. Identification of DEGs

As shown in Figure 3(a), compared to the control, 337 up- and 406 downregulated DEGs were identified in the model group. Furthermore, we found a total of 258 gene changes: 158 DEGs were upregulated and 100 DEGs were downregulated between the BBR (150 mg/kg) and model groups (Figure 3(b)). In addition, 427 DEGs were found between the BBR (300 mg/kg) and model groups (Figure 3(c)), including 280 upregulated and 147 downregulated DEGs. Combining the absolute log2 (Con/DM) ≥ 1 and log2 (BBR/DM) ≥ 1 with the *P* value < 0.05, 55 upregulated and 27 downregulated DEGs were shown with a heat map in the 150 mg/kg BBR group (Figure 3(d)). Also, 91 upregulated and 63 downregulated DEGs were found in the 300 mg/kg BBR group (Figure 3(e)).

### 3.4. Function Enrichment Analysis of DEGs in Rats

Further enrichment analysis is aimed at investigating the biological function of DEGs. As shown in Table S2, the DEGs of the low-dose BBR group with regard to the biological process (BP) were significantly enriched in proteolysis, regulation of calcineurin-NFAT signaling cascade. For the GO molecular function (MF), the DEGs were significantly enriched in single/double-stranded RNA binding, poly(A) RNA binding. For the GO cell component (CC), the DEGs were closely related to extracellular exosome and chromosome. Additionally, KEGG analysis proved that the DEGs were significantly enriched in the PI3K-AKT signaling pathway. Functional annotation of DEGs in the high-dose BBR group is involved in processes in the metabolic process, cellular component organization or biogenesis, and extracellular region [25].

### 3.5. PPM1B Was Upregulated in the Liver of ZDF Rats

**Figure 4:**
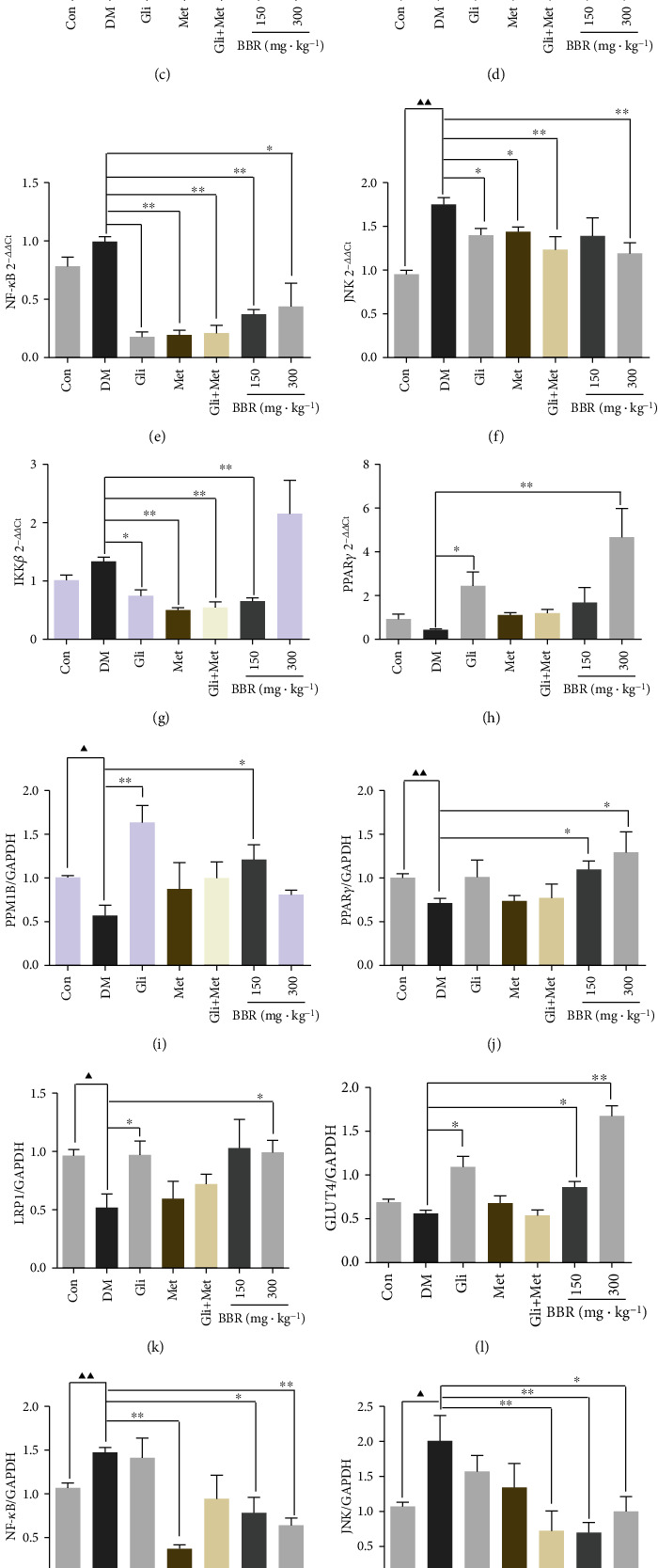
PPM1B was upregulated in the liver of ZDF rats. PPM1B, PPAR*γ*, LRP1, GLUT4, NF-*κ*B, JNK, and IKK*β* mRNA (a) and protein (b) relative expression with medication in ZDF rats. ^▲^*P* < 0.05 and ^▲▲^*P* < 0.01, Con vs. DM; ^∗^*P* < 0.05 and ^∗∗^*P* < 0.01, DM vs. experimental groups.

Based on the microarray analysis, we found that PPM1B expression was upregulated by BBR. To investigate the characteristics of PPM1B in ZDF rats, the mRNA and protein expression levels of PPM1B as well as PPAR*γ*, LRP1, GLUT4, NF-*κ*B, JNK, and IKK*β* were measured by qPCR and Western blotting. As illustrated in Figures 4(a) and 4(b), the mRNA and protein expression levels of PPM1B, PPAR*γ*, LRP1, and GLUT4 in the low- and high-dose BBR groups were dramatically higher than those in DM tissues. In addition, the expression level of NF-*κ*B, JNK, and IKK*β* was significantly downregulated in the metformin and BBR groups.

### 3.6. BBR Prevented Both Glucose Consumption and Uptake Decrease, and the Transcription Levels of Inflammatory Factors Increase in HepG2-IR Cells

**Figure 5:**
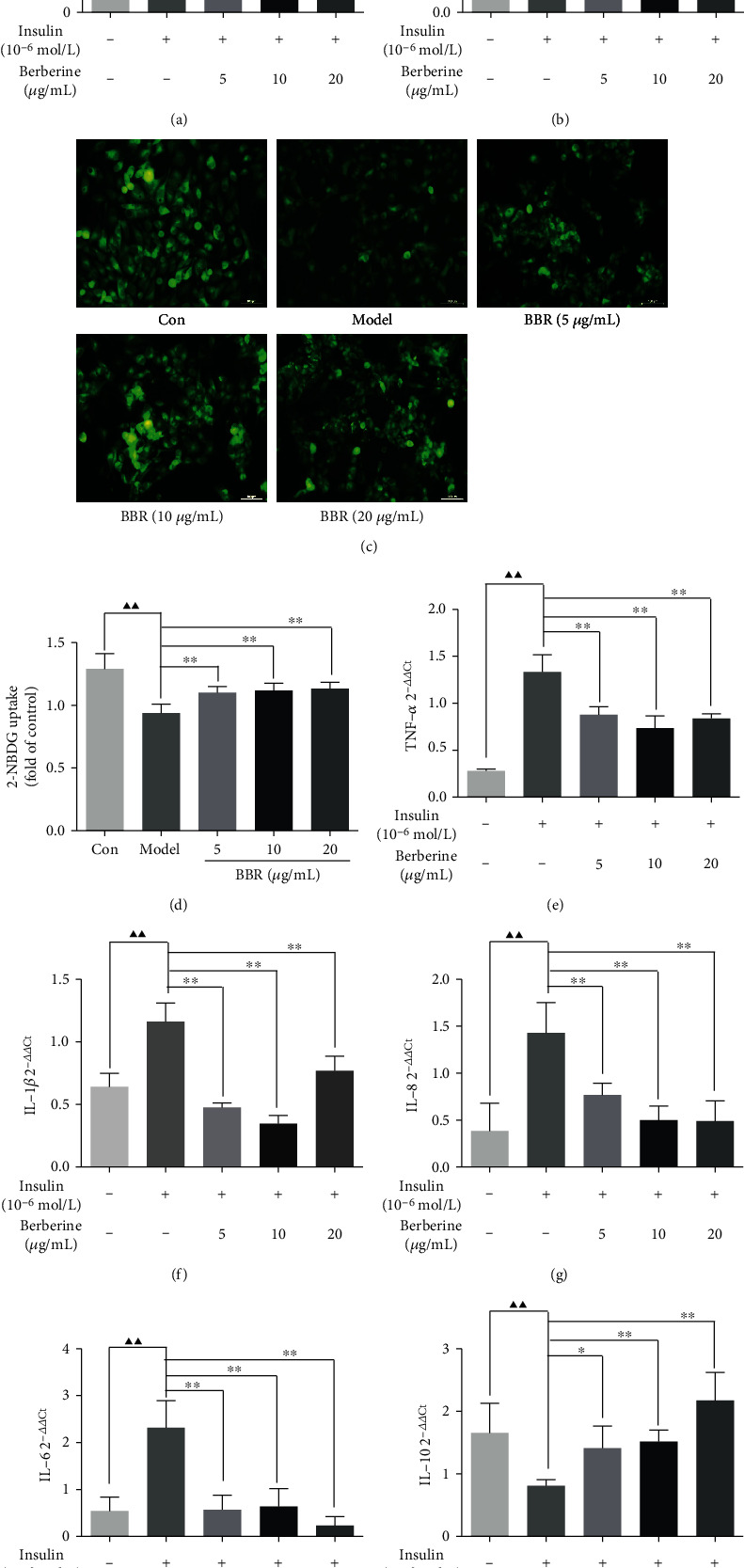
Effect of BBR on glucose consumption, uptake, and inflammatory factors in HepG2-IR cells. (a) Glucose consumption from the culture medium after 24 h treatment. (b) Cell viability of HepG2 cells. (c) Uptake of 2-NBDG into HepG2-IR cells. (d) The quantitative analysis of fluorescence from eight independent replicates. (e–i) The expression of TNF-*α*, IL-1*β*, IL-6, IL-8, and IL-10 in HepG2-IR cells was measured by qPCR. ^▲^*P* < 0.05 and ^▲▲^*P* < 0.01, Con vs. model; ^∗^*P* < 0.05 and ^∗∗^*P* < 0.01, model vs. BBR-treated groups.

Glucose consumption and uptake experiments were performed. In Figures 5(a)–5(d), the glucose consumption and uptake were observably increased by BBR administration at 5, 10, and 20 *μ*g/mL. Furthermore, the TNF-*α*, IL-1*β*, IL-6, IL-8, and IL-10 expression levels were detected via qRT-PCR trial. We observed that BBR reduced the TNF-*α*, IL-1*β*, IL-6, and IL-8 levels (Figures 5(e)–5(h)) while increased the IL-10 level in a dose-dependent fashion in HepG2-IR model cells (Figure 5(i)).

### 3.7. Effect of BBR on the cAMP/PKA/PPM1B Pathway in HepG2-IR Cells

**Figure 6:**
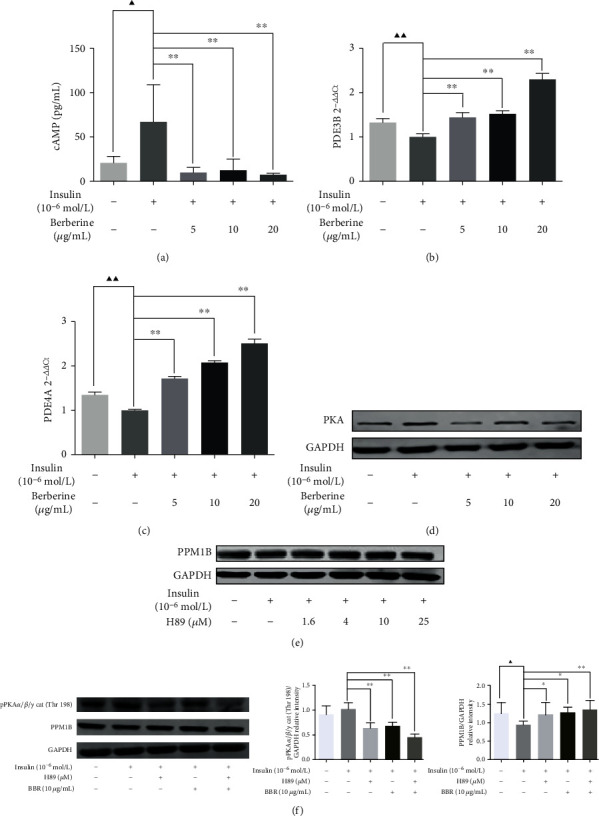
Effect of BBR on the PPM1B/cAMP/PKA pathway in HepG2-IR cells. (a) The content of cAMP measured by the ELISA kit. (b) PDE3B and (c) PDE4A mRNA expression evaluated with qPCR. (d) PKA, (e) PPM1B, and (f) phosphorylated PKA protein levels in total HepG2-IR cell extracts. ^▲^*P* < 0.05 and ^▲▲^*P* < 0.01, Con vs. model; ^∗^*P* < 0.05 and ^∗∗^*P* < 0.01, model vs. H89/BBR-treated groups.

To explore the potential upstream mechanisms, we examined the effect of BBR on the cAMP, PDE3B, and PDE4A expression, which was also combined with H89 to observe the PKA and PPM1B protein expression in HepG2-IR cells. In the present study, we hypothesize that the cAMP/PKA pathway acted as an upstream pathway of PPM1B. As shown in Figure 6(a), the level of cAMP was memorably restrained by BBR. In addition, qRT-PCR outcomes revealed that BBR notably quickened PDE3B and PDE4A expression with concentration dependence (Figures 6(b) and 6(c)) and obstructed PKA expression (Figure 6(d)). In addition, PPM1B expression of HepG2-IR cells was significantly increased at the dosage of 1.6, 4, 10, and 25 *μ*M H89 (Figure 6(e)). Further, H89 combined with BBR treatment exhibited a dramatic downregulation of pPKA and also significantly increased PPM1B protein expression (Figure 6(f)). The findings illustrated that BBR thwarted the cAMP/PKA pathway and contributed to promoting PPM1B levels.

### 3.8. Effect of BBR on the PPM1B/GLUT4 and PPM1B/IKK*β*/NF-*κ*B Pathways in HepG2-IR Cells

**Figure 7:**
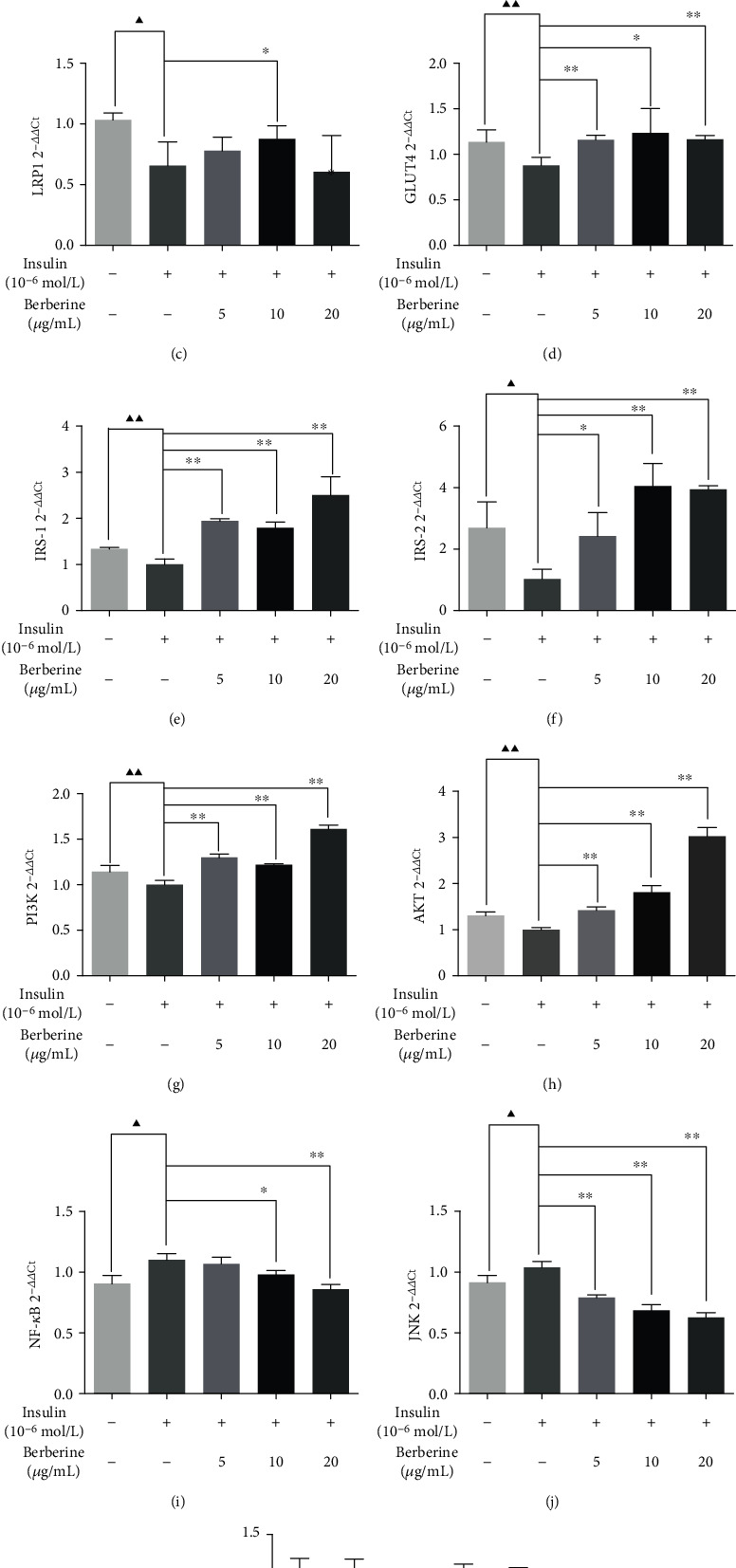
Effect of BBR on the mRNA expression level of targets in the PPM1B pathway in HepG2-IR cells. HepG2 cells without treatment served as a model group. The experiments were repeated three times and presented as mean ± SD. ^▲^*P* < 0.05 and ^▲▲^*P* < 0.01, Con vs. model; ^∗^*P* < 0.05 and ^∗∗^*P* < 0.01, model vs. BBR-treated groups.

**Figure 8:**
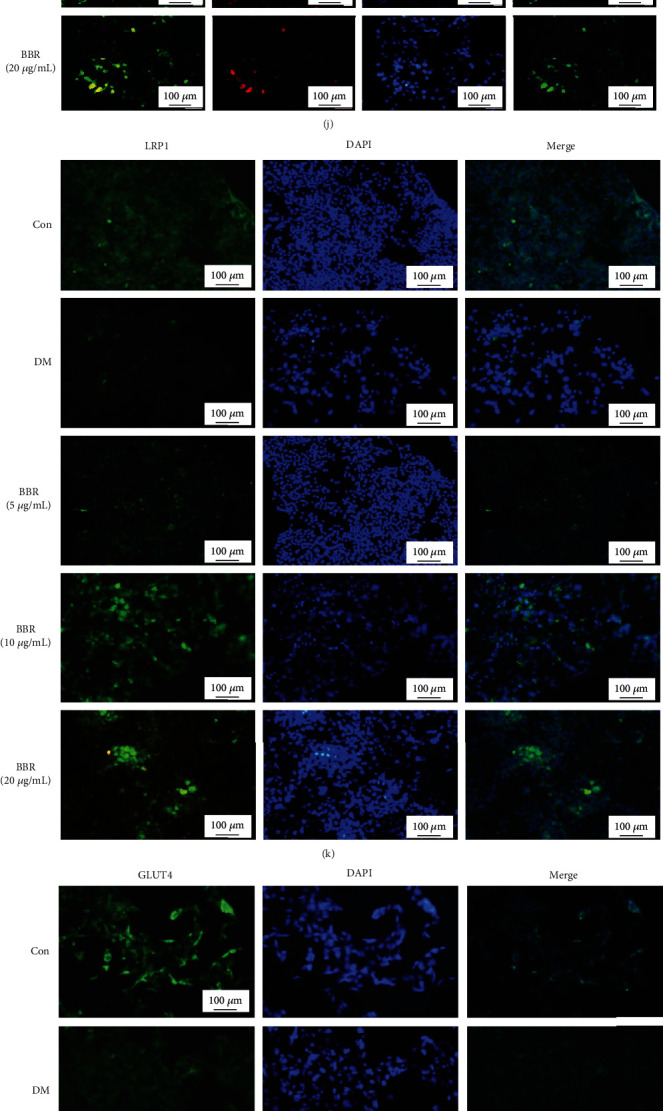
Effect of BBR on the protein expression level of targets in the PPM1B pathway in HepG2-IR cells. (a) Representative Western blotting results of targets. (b–i) Semiquantitative analysis of PPM1B, LRP1, pIKK*β* Ser181, GLUT4, PPAR*γ*, IKK*β*, NF-*κ*B p65, and JNK in liver tissues. The experiments were repeated three times and presented as mean ± SD. ^▲^*P* < 0.05 and ^▲▲^*P* < 0.01, Con vs. model; ^∗^*P* < 0.05 and ^∗∗^*P* < 0.01, model vs. BBR-treated groups. The HepG2-IR cells under the indicated treatment were costained by (j) PPM1B (green) and PPAR*γ* (red) immunofluorescence antibody, as well as (k) LRP1 (green), (l) GLUT4 (green), (m) IKK*β* (green), and (n) NF-*κ*B p65 (green). DAPI (blue) was used to visualize nuclei. Representative microscopic pictures are shown. Magnification, ×100. Scale bar, 100 *μ*m. The experiments were replicated at least three times.

Similarly, we assumed that the PPM1B/GLUT4 pathway, PPM1B/IKK*β*/NF-*κ*B pathway, and PPM1B/PI3K/AKT pathway as the potential downstream are associated with PPM1B. The results showed that PPM1B, PPAR*γ*, LRP1, GLUT4, IRS-1, IRS-2, PI3K, and AKT expression significantly increased in HepG2-IR cells with BBR treatment, respectively (Figures 7(a)–7(h)). Additionally, both the NF-*κ*B p65 and JNK mRNA expression levels were lower than the model group (Figures 7(i) and 7(j)), and there was no obvious difference in IKK*β* expression (Figure 7(k)). The results of Western blot showed that PPM1B and LRP1 were markedly increased in the BBR-treated groups (Figures 8(a)–8(c)). Phosphorylation of Ser181 plays an important role in the activation of the catalytic activity of IKK.  Figure 8(d) indicates that BBR produced a distinct reduction in the value of pIKK*β* Ser181/total IKK*β* (Figure 8(d)). In addition, GLUT4 and PPAR*γ* protein expression levels were significantly higher in the BBR groups (Figures 8(e) and 8(f)), and there was no significant difference in the total protein of IKK*β* (Figure 8(g)). The NF-*κ*B p65 and JNK expression levels were significantly downregulated compared to IR cells (Figures 8(h) and 8(i)). To further confirm the expression of PPM1B pathways, immunofluorescence staining was performed. It showed that the expression levels of PPM1B and PPAR*γ* in the cells treated with BBR were synchronously augmented (Figure 8(j)). Immunofluorescence data of LRP1, GLUT4, IKK*β*, and NF-*κ*B p65 shown in Figures 8(k)–8(n) are consistent with the results of Western blot.

### 3.9. Effect of BBR on the PPM1B/PI3K/AKT Pathway in HepG2-IR Cells

**Figure 9:**
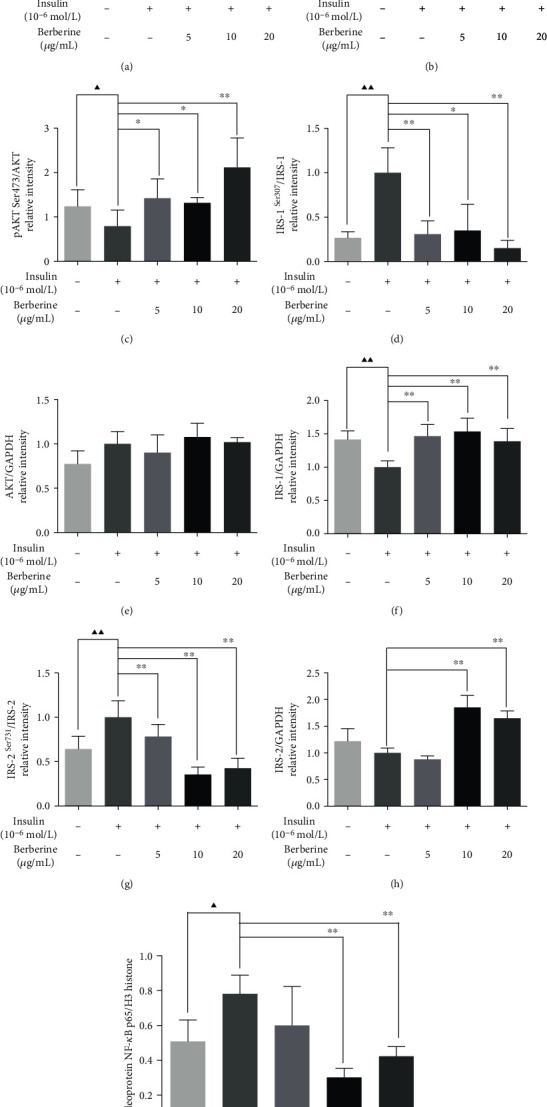
Effect of BBR on the PI3K/AKT pathway in HepG2-IR cells. (a) Representative Western blotting results of PI3K p85, pAKT Ser473, AKT, pIRS-1 Ser307, IRS-1, pIRS-2 Ser731, IRS-2, and nucleoprotein NF-*κ*B p65. (b–i) Semiquantitative analysis of PI3K p85, pAKT Ser473, AKT, pIRS-1 Ser307, IRS-1, pIRS-2 Ser731, IRS-2, and nucleoprotein NF-*κ*B p65. The experiments were repeated three times and presented as mean ± SD. ^▲^*P* < 0.05 and ^▲▲^*P* < 0.01, Con vs. model; ^∗^*P* < 0.05 and ^∗∗^*P* < 0.01, model vs. BBR-treated groups.

For the PPM1B/PI3K/AKT pathway, as shown in Figures 9(a) and 9(b), the pIRS-1 Ser307 and pIRS-2 Ser731 protein expression markedly reduced, respectively, while IRS-1, IRS-2, pAKT Ser473, and PI3K p85 show significant upregulation. Interestingly, there was no difference of AKT in cells. The NF-*κ*B nuclear translocation is activated by IKK*β* that would be conducive to inflammatory responses. The expression of nucleoprotein NF-*κ*B p65 was analyzed by Western blot. In addition, the protein of NF-*κ*B p65 was significantly downregulated in the BBR groups. The results showed that the PPM1B-related pathway could participate in the regulation of glucose metabolism, inflammation, and activation of the insulin signaling cascade.

### 3.10. Effect of Silenced PPM1B in HepG2-IR Cells

**Figure 10:**
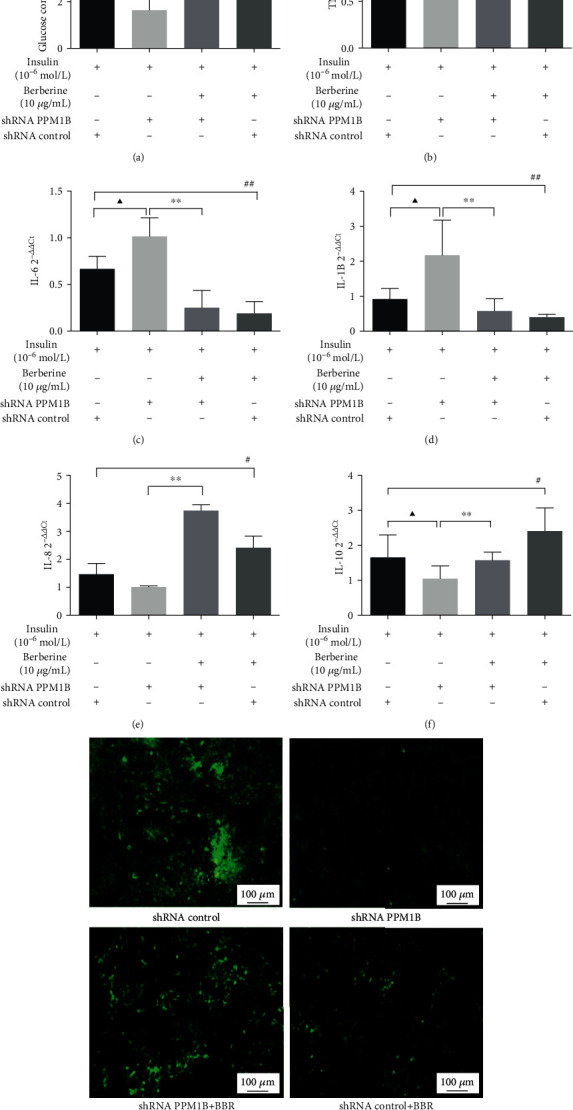
Silenced PPM1B promoted glucose consumption and inflammatory cytokine levels and weakened glucose uptake capacity in HepG2-IR cells. (a) Glucose consumption of unit cell. (b) TNF-*α* levels. (c) IL-6 levels. (d) IL-1B levels. (e) IL-8 levels. (f) IL10 levels. (g) 2-NBDG uptake (2-(N-(7-nitrobenz-2-oxa-1,3-diazol-4-yl)amino)-2-deoxyglucose). The data are presented as mean ± SD of three independent experiments. ^▲^*P* < 0.05 and ^▲▲^*P* < 0.01, shRNA control group vs. shRNA PPM1B group; ^∗^*P* < 0.05 and ^∗∗^*P* < 0.01, shRNA PPM1B+BBR group vs. shRNA PPM1B group; ^#^*P* < 0.05 and ^##^*P* < 0.01, shRNA control+BBR group vs. shRNA control group. Magnification, ×100. Scale bar, 100 *μ*m.

**Figure 11:**
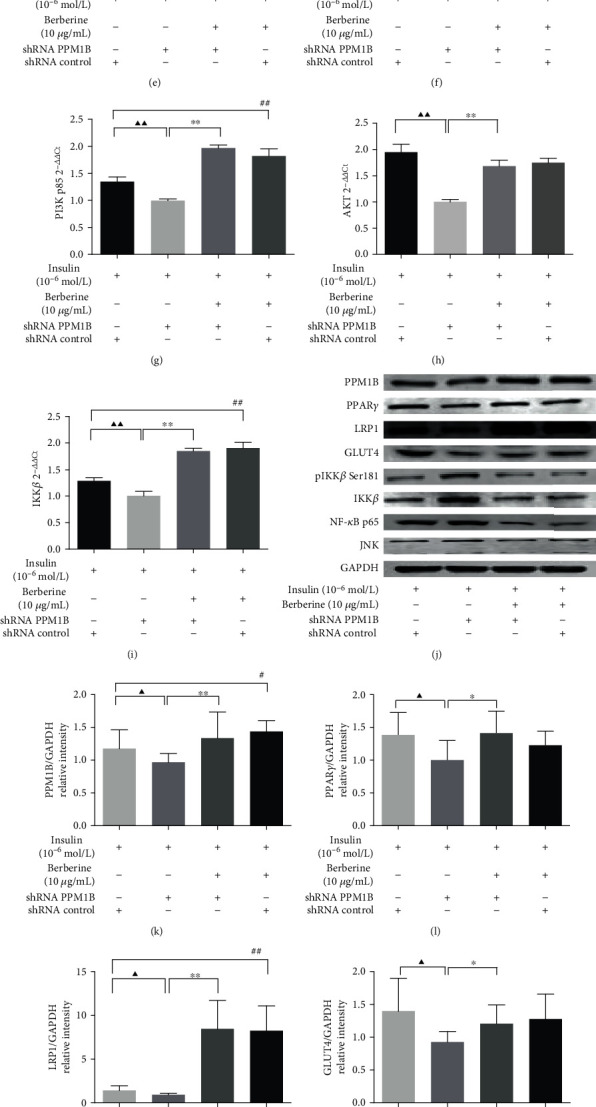
Effect of BBR on the relative expression of PPM1B, PPAR*γ*, LRP1, GLUT4, IRS-1, IRS-2, PI3K, AKT, pIKK*β* Ser181, IKK*β*, NF-*κ*B p65, and JNK in HepG2-IR cells with silenced PPM1B by lentivirus. GAPDH was utilized as an internal reference. Data were obtained by measuring the gray value of each bar from the result of Western blotting and were presented as the fold change relative to that of GAPDH. ^▲^*P* < 0.05 and ^▲▲^*P* < 0.01, shRNA control group vs. shRNA PPM1B group; ^∗^*P* < 0.05 and ^∗∗^*P* < 0.01, shRNA PPM1B+BBR group vs. shRNA PPM1B group; ^#^*P* < 0.05 and ^##^*P* < 0.01, shRNA control+BBR group vs. shRNA control group.

To determine the function of PPM1B in IR, HepG2-IR cells were transfected with shPPM1B lentivirus. As shown in Figure 10(a), the unit cells with silenced PPM1B displayed weakened glucose consumption, which would be reversed by BBR treatment. Similarly, PPM1B knockdown caused the TNF-*α*, IL-6, and IL-1B expression to significantly rise compared to the control group (Figures 10(b)–10(d)). Contrarily, IL-8 and IL-10 expression levels were lower in the shRNA PPM1B group (Figures 10(e) and 10(f)). Meanwhile, TNF-*α*, IL-6, and IL-1B mRNA expression levels were significantly downregulated while IL-10 was upregulated in shRNA PPM1B HepG2-IR cells. To examine the effect of silenced PPM1B on cell glucose uptake capacity, 2-NBDG assays were performed, and the result revealed that the knockdown of PPM1B significantly decreased glucose uptake capacity of HepG2-IR cells (Figure 10(g)). To determine the underlying mechanism of BBR, the PPM1B, PPAR*γ*, LRP1, GLUT4, IRS-1, IRS-2, PI3K, AKT, and IKK*β* mRNA expression and the PPM1B, PPAR*γ*, LRP1, GLUT4, pIKK*β* Ser181, IKK*β*, NF-*κ*B p65, and JNK protein expression in HepG2-IR cells were assessed under transfection with shPPM1B lentivirus. As shown in Figures 11(a)–11(i), the PPM1B, PPAR*γ*, LRP1, GLUT4, IRS-1, IRS-2, PI3K p85, AKT, and IKK*β* mRNA expression levels in PPM1B knockdown HepG2-IR cells were dramatically lower than those in the shRNA control group. In addition, results from Western blot showed that the PPM1B, PPAR*γ*, LRP1, and GLUT4 protein levels were also significantly suppressed with PPM1B silencing (Figures 11(j)–11(n)). Compared with shPPM1B cells, the pIKK*β* Ser181, IKK*β*, NF-*κ*B p65, and JNK protein expression levels were remarkably downregulated in the shPPM1B+BBR group (Figures 11(o)–11(r)). These in vitro findings indicate that BBR significantly enhances glucose consumption and uptake and reduces inflammation of HepG2-IR cells by upregulating PPM1B expression.

## 4. Discussion

Berberine (BBR), a plant alkaloid isolated from a traditional Chinese herb, has already been used in bacterial infection and dysentery for a hundred years [26]. Accumulating studies certified that BBR has crucial roles in the treatment of metabolic syndrome and IR. To our knowledge, IR is characterized by decreased sensitivity and responsiveness to insulin in peripheral tissues, which results in dyslipidemia, obesity, hypertension, atherosclerosis, liver failure, and cancers. Previous studies suggested that T2D also is a chronic inflammatory disease [27].

TNF-*α*, a part of proinflammatory factors, is involved in the development of obesity-related diseases, which damages the insulin signaling pathway mainly through the NF-*κ*B p65 and JNK inflammatory transduction pathways. Previous studies have indicated that chronic low-grade inflammation in liver tissue plays a key role in T2D [28, 29]. Our findings also found that BBR might be considered a potential agent for the regulation of glucose metabolism by reducing blood glucose levels. In vitro experiments showed that the expression of proinflammatory cytokines in the HepG2 cells was inhibited by BBR, which indicated that BBR may moderate the low-grade inflammatory response in IR. To observe whether BBR attenuated the glucose metabolism and inflammatory response via the PPM1B pathway, we used shRNA PPM1B to knock down PPM1B expression in HepG2-IR cells. Knockdown of PPM1B in insulin-stimulated HepG2 cells displayed a greater inflammatory state and dysfunction of glycometabolism, while the effects could be partially reversed by BBR through upregulating the mRNA and protein expression levels of PPM1B, PPAR*γ*, LRP1, GLUT4, IRS-1, IRS-2, PI3K, AKT, and IKK*β* and inhibiting the phosphorylation level of pIKK*β* Ser181 as well as total IKK*β*, NF-*κ*B p65, and JNK. Our data preliminarily discussed that PPM1B might play an IR suppressor role in T2D, serving as a downstream target of cAMP/PKA signaling, thus regulating metabolic disorder.

**Figure 12:**
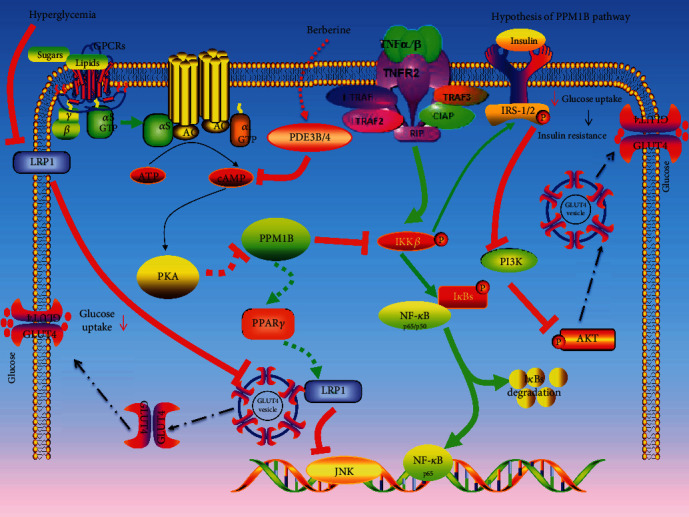
PPM1B interacts with IKK*β* through which it regulates activation of NF-*κ*B. LRP1 interacts with JNK and NF-*κ*B through which it regulates gene expression of JNK and NF-*κ*B. Hyperglycemia suppresses the levels of LRP1, which might further impair glucose homeostasis in the liver. LRP1 plays an important role in the regulation of glucose homeostasis by controlling glucose transporter levels. Activation of IKK*β* via the phosphorylation of Ser181 in IKK*β*, a serine phosphorylated kinase of the insulin receptor, contributing to phosphorylation of Ser307 in IRS-1 and Ser731 in IRS-2 to block insulin signal transduction to PI3K.

It is essential to explore the underlying mechanisms of IR to ameliorate the inflammatory response for patients with T2D. Consistent with the previous results, BBR could facilitate HepG2-IR cells' glucose uptake and consumption properties by regulating multidimensional anti-inflammatory mechanisms [30]. As shown in Figure 12, accumulating evidence claims that G Protein-Coupled Receptors (GPCRs), a class of membrane protein receptors, characterized by seven-transmembrane *α* helix in their stereoscopic structures, binds to the carbohydrates, lipids, and polypeptides in the surrounding environment of cells to change the signal transduction. With the stimulation of the external environment, the extracellular signaling molecule firstly binds to the receptor to form a complex, then activates the stimulatory G*α* protein (Gs*α*) on the cell membrane, leading to an increase in intracellular cAMP and activation of PKA. In contrast, activation of phosphodiesterase leads to termination of cAMP signal transduction, which in turn decreases cAMP levels [31]. In this study, we hypothesized that BBR contributes to the transformation of cAMP into AMP by promoting phosphodiesterase expression and participating in termination of cAMP/PKA signaling. Consistently, activation of PDE4B inhibits the cAMP/PKA pathway mediated by glycogen [32]. As we expected, BBR decreased the level of cAMP and increased the PDE3B and PDE4A expression in HepG2-IR cells. Additionally, Choi et al. demonstrated that PKA destabilizes PPM1B upon inflammatory stimuli via phosphorylation of Ser195 in PPM1B [33]. Furthermore, PKA was inhibited by H89, demonstrating that H89 treatment efficiently blocked the decrease of PPM1B protein expression in the current study. Importantly, the previous study reported that PPM1B blocks the nuclear translocation of NF-*κ*B by IKK*β* inhibition, which consequently reduced the expression of inflammatory factors [34–36]. Thus, the PPM1B/IKK*β*/NF-*κ*B pathway may be a therapeutic strategy for IR in T2D. In addition, the previous study indicated that PPM1B indirectly increases PPAR*γ* expression [37]. PPAR*γ* is the target of thiazolidinedione that is associated with adipocyte differentiation, immunity, and IR. Wang et al. also demonstrated that PPAR*γ* agonists increase LRP1 expression [36]. In addition, LRP1, a major apolipoprotein E receptor, inhibits the JNK and NF-*κ*B pathways to reduce the production of inflammatory factors for lipid metabolism. Further, LRP1 is identified as a major component of GLUT4-positive vesicles, which interacts with the lumenal domains of GLUT4 [37, 38]. Liu et al. demonstrated that hyperglycemia suppresses the level of LRP1, which is related to the regulation of brain glucose homeostasis by controlling the glucose transporter and insulin signaling [38]. Hyperglycemia may downregulate LRP1 expression, which leads to a vicious cycle which decreased the translocation of GLUT4 to the plasma membrane. Interestingly, our study indicated that PPM1B may be reversed by the reduction of LRP1 by BBR. Meanwhile, activation of IKK*β* via the phosphorylation of IKK*β* on Ser181, contributing to phosphorylation of Ser307 in IRS-1 and Ser731 in IRS-2, then blocks insulin signal transduction to PI3K [20, 39]. Together with previous studies, our results seem to point out that PPM1B silencing in HepG2-IR cells has potential to suppress the glucose uptake, consumption, and insulin signal transduction. However, there are several limitations in our study. Specifically, the accurate relationship of upstream and downstream of PPM1B and the effects of PPM1B in T2D rats remain to be explored. Thus, further assays are needed to investigate the functions of PPM1B in vivo and in vitro.

## 5. Conclusion

In summary, we demonstrated that BBR not only ameliorated glucose metabolism and insulin signal transduction on HepG2-IR cells through upregulating PPM1B levels, and attenuated the stimulation of high glucose by decreasing cAMP/PKA signaling, but also suppressed the inflammation under IR. The anti-IR mechanism of BBR might be correlated with the regulation on the PPM1B signaling pathway, including the cAMP/PKA/PPM1B, PPM1B/GLUT4, PPM1B/IKK*β*/NF-*κ*B, and PPM1B/PI3K/AKT pathways. Therefore, we concluded that BBR can effectively protect T2D rats against IR by regulating the expression of cAMP, PKA, PPM1B, PPAR*γ*, LRP1, GLUT4, NF-*κ*B p65, JNK, IKK*β*, IRS-1, IRS-2, PI3K, and AKT in the liver.

## Data Availability

The datasets analyzed in the current study are available from the corresponding author on reasonable request.

## Supplementary Materials

Supplementary Materials Supplementary Figure S1: effects of insulin on the cell viability, glucose consumption, and glucose uptake in HepG2 cells. Supplementary Figure S2: effects of BBR on the HepG2 cell viability. Supplementary Figure S3: efficient PPM1B knockdown at the mRNA and protein levels in HepG2 cells using lentiviral-mediated RNAi. Supplementary Table S1: primers of qPCR. Supplementary Table S2: functional and pathway enrichment analysis of DEGs in the low-dose BBR group.
