# Supplementary material for: Berberine Improves Inflammatory Responses of Diabetes Mellitus in Zucker Diabetic Fatty Rats and Insulin-Resistant HepG2 Cells through the PPM1B Pathway
Source: J Immunol Res. 2020 Aug 18;2020:2141508. doi: 10.1155/2020/2141508 (PMC7450322; doi:10.1155/2020/2141508)
Supplement: Supplementary Materials — Supplementary Figure S1: effects of insulin on the cell viability, glucose consumption, and glucose uptake in HepG2 cells. Supplementary Figure S2: effects of BBR on the HepG2 cell viability. Supplementary Figure S3: efficient PPM1B knockdown at the mRNA and protein levels in HepG2 cells using lentiviral-mediated RNAi. Supplementary Table S1: primers of qPCR. Supplementary Table S2: functional and pathway enrichment analysis of DEGs in the low-dose BBR group. [file 2141508.f1.pdf]

Supplementary Figure Legends

Supplementary Figure 1S Effects of BBR on the HepG2 cell viability. The cell viability was measured by MTT with BBR treatment at a dose of 0, 5, 10, 20, 30, 40, and 50 μg/ml for 24h (A) and 48h (B). **P<0.05* and ***P<0.01* BBR-treated groups *vs* Control group.

Supplementary Figure 2S Effects of insulin on the cell viability, glucose consumption and glucose take-up in HepG2 cells. The cell viability was measured by MTT with insulin treatment at a dose of 10^{-5}, 10^{-6}, 10^{-7} and 10^{-8} mol/L for 24h (A) and 48h (B). (C) The glucose consumption was significantly decreased with 10^{-5} or 10^{-6} mol/L insulin treatment. (D) The fall range of glucose consumption was bigger in 36 h after treatment with insulin at a dose of 10^{-6} mol/L. (E) Representative images of glucose take-up in HepG2 cells. (F) The quantitative analysis of fluorescence associated with 2-NBDG take-up. **P<0.05* and ***P<0.01* Insulin groups vs Control group.

Supplementary Figure 3S Efficient PPM1B knockdown at the mRNA and protein levels in human HepG2 cells using lentiviral-mediated RNAi. (A) Representative images of GFP expression in HepG2 cells after infection using shNC or shPPM1B-1/PPM1B-2/PPM1B-3 lentiviruses. PPM1B mRNA (B) and protein(C) expression in HepG2 cells after transfection of shNC or shPPM1B-1 /PPM1B-2 /PPM1B-3 lentiviruses. GAPDH was used as an internal control, and the data represent the mean ± SD of three independent experiments. (**P<0.05*, ***P < 0.01;* shPPM1B-1/PPM1B-2/PPM1B-3 lentiviruses vs shNC group; Magnification,×100; Scale bar, 100μm)

Supplementary Figure 1S

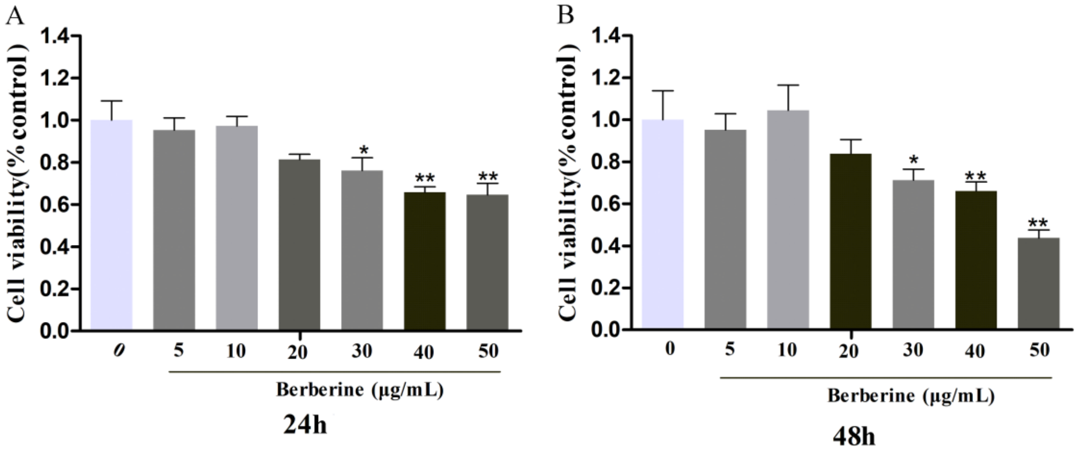

Supplementary Figure 2S

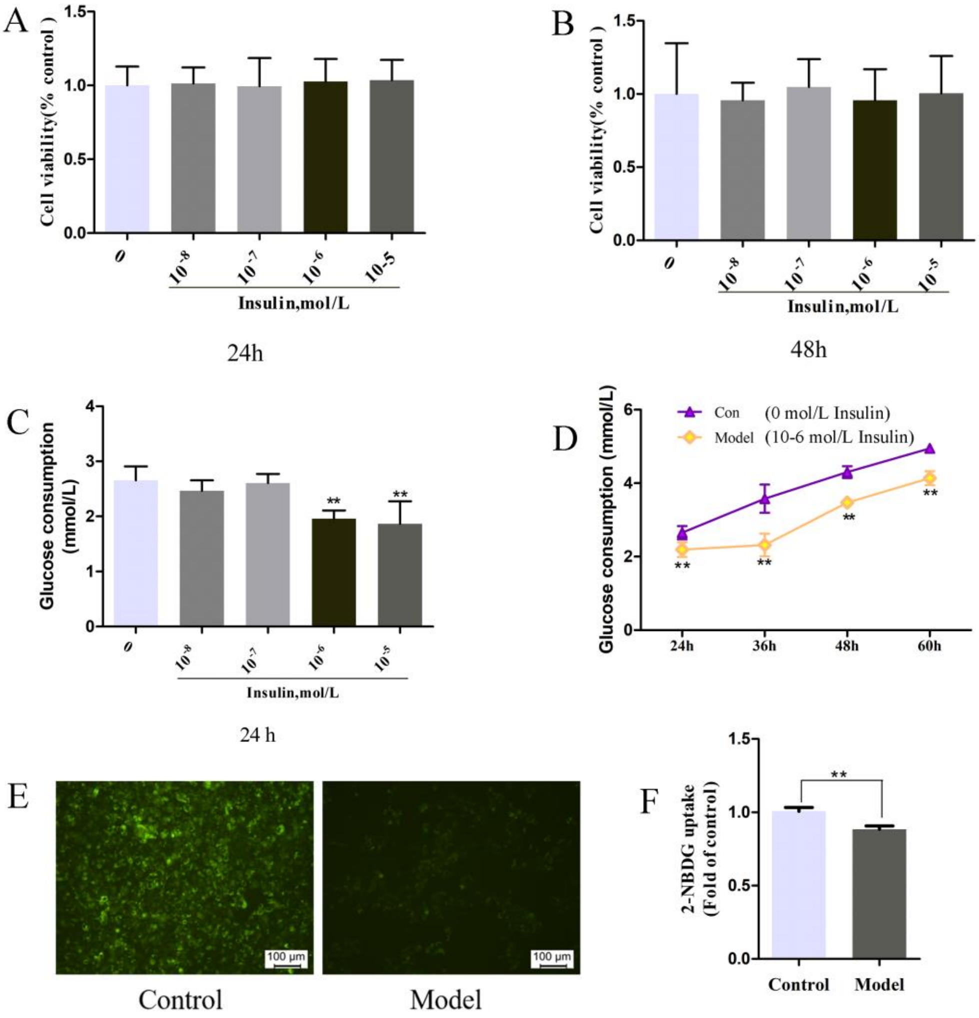

Supplementary Figure 3S

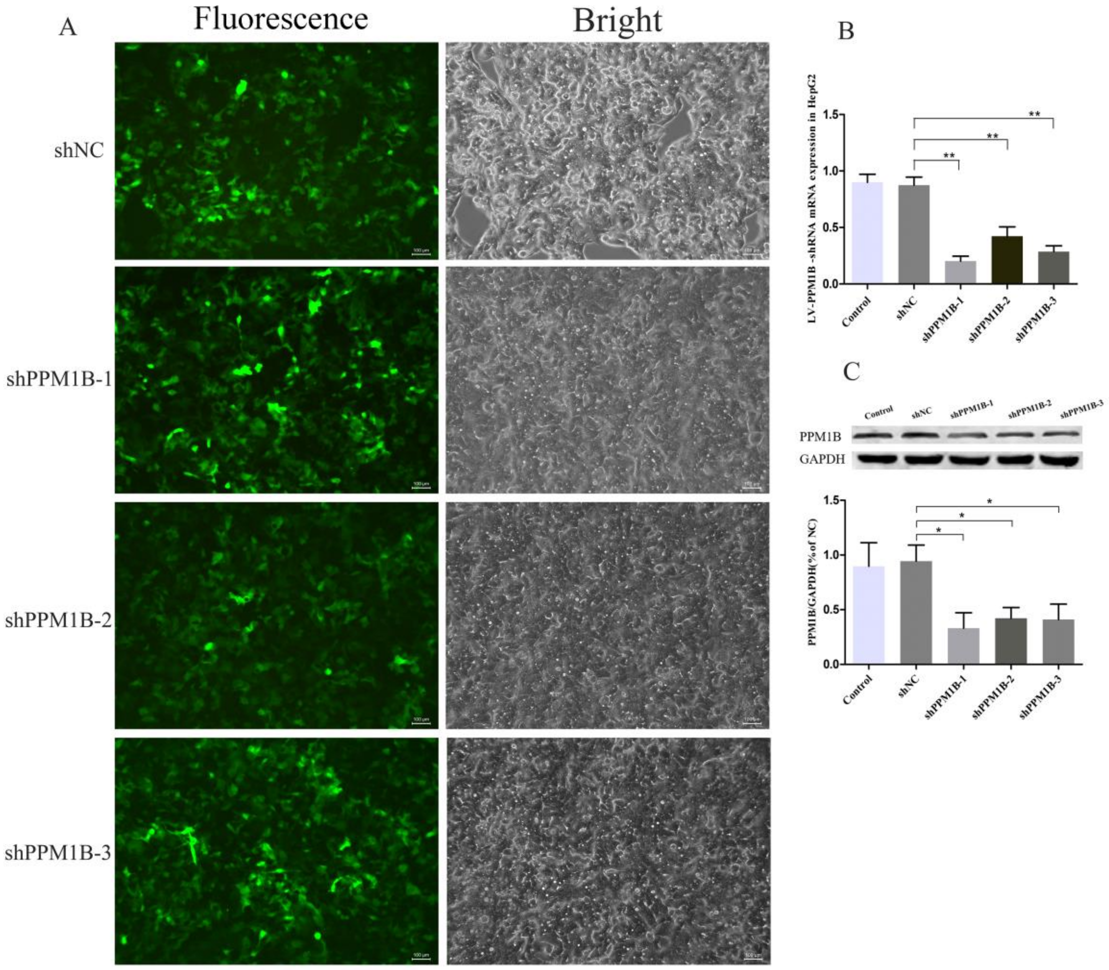

Table S1 Primer of q-PCR

Primer	Species	Sequence 5'-3'
TNFα-F	Homo sapiens	AGGACCAGCTAAGAGGGAGA
TNFα-R	Homo sapiens	CCCGGATCATGCTTTCAGTG
IL-1β-F	Homo sapiens	ATGCCACCTTTTGACAGTGATG
IL-1β-R	Homo sapiens	AGCTTCTCCACAGCCACAAT
IL-6-F	Homo sapiens	AAGCCAGAGTCATTCAGAGCAATACTG
IL-6-R	Homo sapiens	GATGAGTTGGATGGTCTTGGTCCTTAG
IL-8-F	Homo sapiens	TTGGCAGCCTTCCTGATTTCT
IL-8-R	Homo sapiens	TTTGGGGTGGAAAGGTTTGGA
IL-10-F	Homo sapiens	GCCTTCAGCAGAGTGAAGACT
IL-10-R	Homo sapiens	GGCAACCCAGGTAACCCTTA

PDE3B-F	Homo sapiens	AATGGCCCAGCAAAAGTTCG
PDE3B-R	Homo sapiens	GAGTTACACAGGGGACCCAC
PDE4A-F	Homo sapiens	GCGGGACCTACTGAAGAAATTCC
PDE4A-R	Homo sapiens	CAGGGTGGTCCACATCGTGG
PPM1B-F	Homo sapiens	GCTTGTGATGGGATCTGGGA
PPM1B-R	Homo sapiens	TCTCCATAATCTCTTCAACCCGT
IKKβ-F	Homo sapiens	AGCTCTGGAACCTCCTGAAGA
IKKβ-R	Homo sapiens	AGCTCCAGTCTAGGGTCGTGA
PPARγ-F	Homo sapiens	CTGCCGGATCCACAAAAAAAG
PPARγ-R	Homo sapiens	CGCCAACAGCTTCTCCTTCTCG
LRP1-F	Homo sapiens	TCCTGCCTGCTCCTCAGTAT
LRP1-R	Homo sapiens	AGAGCCAAGGAAAGGAAAGC
NFκB-F	Homo sapiens	AGTTGAGGGGACTTTCCCAGGC
NFκB -R	Homo sapiens	GCCTGGGAAAGTCCCCTCAACT
JNK1-F	Homo sapiens	GTGGGGTATGCCCAAGAGG
JNK1-R	Homo sapiens	GCCATAAAGCCCAGATAGAGC
GLUT4-F	Homo sapiens	CAGCTCTCAGGCATCAAT
GLUT4-R	Homo sapiens	TCTACTAAGAGCACCGAG
IRS1-F	Homo sapiens	CAAGAGCATGCACAAACGCT
IRS1-R	Homo sapiens	GCCCGCTTGTTGATGTTGAA
PI3K p85-F	Homo sapiens	TCCTGCTATGGCAGACATCC
PI3K p85-R	Homo sapiens	AGAAATGGTAGCTTCCCGAGG
AKT-F	Homo sapiens	TCGGAGACTGACACCAGGTA
AKT-R	Homo sapiens	CTGGCCGAGTAGGAGAACTG
IRS2-F	Homo sapiens	CTCACCCTGTAGTGCCTTCG
IRS2-R	Homo sapiens	ATGTTGATGTACTCGCCGGG
GAPDH-F	Homo sapiens	CGGGGCTGGCATTGCTCTC
GAPDH-R	Homo sapiens	GGGGCCGACTTGGGATAGG

Table S2 Functional and pathway enrichment analysis of DEGs in BBR (150mg/Kg) group

Category	Term	Description	Genes	FDR
GO_BP	GO:0006508	proteolysis	Capn11,Casp14,Ctsd ,Cma1, Lonp1, Mst1,Sri	7.4E-3
GO_BP	GO:0070884	regulation of calcineurin-NFAT signaling cascade	Nfat5, Rcan1	1.8E-2
GO_BP	GO:0050771	negative regulation of axonogenesis	Mbp, Pten	4.6E-2
GO_MF	GO:0042802	identical protein binding	Rbmx, Lyc2, Mri1, Pten, Rcan1, Safb2, Sri, Trip10, Tfrc	3.6E-3
GO_MF	GO:0003727	single-stranded RNA binding	Rbmx, Hmgb1, Lonp1	1.3E-2
GO_MF	GO:0003725	double-stranded RNA binding	Hsp90ab1, Hmgb1, Tfrc	3.0E-2
GO_MF	GO:0003723	poly(A) RNA binding	Rbmx,Fam98a,Hsp90ab1,Hmgb1,Nol8, Ppan, Rps12, Safb2, Tfrc, Zcchc9	3.1E-2
GO_MF	GO:0000166	nucleotide binding	Rbmx, Marf1, Nol8, Rcan1, Safb2	3.7E-2
GO_MF	GO:0042277	peptide binding	Ctsd, Cma1, Hmgb1	3.8E-2
GO_CC	GO:0070062	extracellular exosome	Gng5,Lypd2,Rbmx,Arpc1a,Asna1, Casp14, Ctsd, Hsp90ab1, Lyc2, Mst1, Pgm2, Safb2, Sri, Tnxb, Trip10, Tfrc, Zfp445	2.1E-2
GO_CC	GO:0005694	chromosome	Recql, Setd2, Hmgb1	3.0E-2
GO_CC	GO:0043231	Intracellular membrane-bounded organelle	Btaf1, Ahcyl2, Cma1, Safb2, Snapc1, Trip10, Tfrc	3.6E-2
GO_CC	GO:0042470	melanosome	Ctsd, Hsp90ab1, Tfrc	4.1E-2
GO_CC	GO:0042995	cell projection	Mbp, Pten, Trip10	4.6E-2
KEGG pathway	hsa04151	PI3K-Akt signaling pathway	Gng5, Atf6b, Hsp90ab1, Lamb3, Pten	3.2E-2

Abbreviations: GO, gene ontology; KEGG, Kyoto Encyclopedia of Genes and Genomes.BP, biological process; CC, cellular component; MF, molecular functions; FDR, false discovery rate
